# Identification and characterization of transition metal-binding proteins and metabolites in the phloem sap of *Brassica napus*

**DOI:** 10.1016/j.jbc.2024.107741

**Published:** 2024-08-31

**Authors:** Hendrik Küpper, Arun Gokul, Dario Alavez, Singha R. Dhungana, Syed Nadeem Hussain Bokhari, Marshall Keyster, David G. Mendoza-Cozatl

**Affiliations:** 1 Czech Academy of Sciences, Biology Centre, Institute of Plant Molecular Biology, Laboratory of Plant Biophysics and Biochemistry, České Budějovice, Czech Republic; 2 Department of Experimental Plant Biology, University of South Bohemia, České Budějovice, Czech Republic; 3 Department of Plant Sciences, Qwaqwa Campus, University of the Free State, Phuthaditjhaba, South Africa; 4 Division of Plant Sciences, University of Missouri-Columbia, Columbia, Missouri, USA; 5 Division of Biological Sciences, University of Missouri-Columbia, Columbia, Missouri, USA; 6 Environmental Biotechnology Laboratory, Department of Biotechnology, University of the Western Cape, Bellville, South Africa

**Keywords:** glutathione, metallothioneins, size exclusion chromatography, phloem sap, iron, zinc

## Abstract

Transition metal (TM) distribution through the phloem is an essential part of plant metabolism and is required for systemic signaling and balancing source-to-sink relationships. Due to their reactivity, TMs are expected to occur in complexes within the phloem sap; however, metal speciation in the phloem sap remains largely unexplored. Here, we isolated phloem sap from *Brassica napus* and analyzed it *via* size exclusion chromatography coupled online to sector-field ICP-MS. Our data identified known TM-binding proteins and molecules including metallothioneins (MT), glutathione, and nicotianamine. While the main peak of all metals was low MW (∼1.5 kD), additional peaks ∼10 to 15 kD containing Cu, Fe, S, and Zn were also found. Further physicochemical analyses of MTs with and without affinity tags corroborated that MTs can form complexes of diverse molecular weights. We also identified and characterized potential artifacts in the TM-biding ability of *B. napus* MTs between tagged and non-tagged MTs. That is, the native BnMT2 binds Zn, Cu, and Fe, while MT3a and MT3b only bind Cu and Zn. In contrast, his-tagged MTs bind less Cu and were found to bind Co and Mn and aggregated to oligomeric forms to a greater extent compared to the phloem sap. Our data indicates that TM chemistry in the phloem sap is more complex than previously anticipated and that more systematic analyses are needed to establish the precise speciation of TM and TM-ligand complexes within the phloem sap.

Transition metals (TM) such as iron (Fe), zinc (Zn), manganese (Mn), and copper (Cu), are essential nutrients critical for plant growth and development as they act as co-factors for several biological processes including respiration, photosynthesis, nucleic acid synthesis, and primary and secondary metabolism (1, 2, 3). TM are also very reactive elements, and therefore their uptake and allocation within tissues need to be tightly regulated to prevent cell damage *via* specific toxicity mechanisms that depend on the element and the concentration (4, 5). In turn, plants have evolved complex mechanisms to maintain TM homeostasis. In vascular plants, these mechanisms can be divided into five major processes: (i) root uptake and intercellular mobilization, (ii) xylem-loading/unloading (iii) phloem-loading/unloading, (iv) intracellular sequestration, (v) intracellular binding to functional or detoxification/storage molecules (1, 6). At the root level, TM are taken up by transporters located at the root epidermis and partitioned between cell compartments and their target proteins according to the cell’s demand. In higher plants, root-to-shoot translocation of TM is driven by the transpiration stream through the xylem, while re-distribution within the plant including leaf-to-leaf and leaf-to-root, is mediated by the phloem (6, 7). TM are mobilized *via* symplast to the xylem parenchyma where specific transporters load them into the xylem stream for root-to-shoot translocation (1, 8). Once in aerial tissues, cells retrieve TM from the xylem and integrate them into their respective metabolic pathways or store them in vacuoles when there is a surplus; this process is particularly pronounced in metal hyperaccumulators (9). In dicots like *Arabidopsis* and other *Brassicales*, TM need to be re-mobilized from mature leaves to sink tissues (*i.e.*, young leaves, seeds, and roots) *via* the phloem, which is a plant tissue made of two types of cells, companion cells, and sieve elements (10, 11). Companion cells are highly specialized cells that import nutrients and other molecules into the sieve element and generate a solute gradient for long-distance transport between mature leaves, younger leaves, roots, and seeds (12).

In recent years, the role of phloem in long-distance nutrient signaling has gained significant attention, as phloem can re-shape root morphology and physiology based on the nutritional status of leaves (13, 14). For instance, legumes capable of interacting with rhizobia to form nitrogen-fixing nodules rely on long-distance signaling to assess the nitrogen availability within the plant and allow, or prevent, the formation of nodules (15). This feedback communication is critical for the plant’s economy, as nitrogen is often a yield-limiting nutrient, but the formation and establishment of nodules require a significant investment of photosynthates. Moreover, TM originally acquired by the plant have to be diverted to symbiosomes for N_2_-fixating metabolism (16). Similarly, shoot-to-root signaling has also been found to play a major role in TM homeostasis. The current model suggests that leaves sense the levels of Fe, Zn, and possibly other TM, and communicate this information to roots to regulate their uptake according to the TM status of the entire plant (14, 17, 18). To date, the precise chemical nature of the phloem signal regulating root uptake of TM remains unknown; however, *A. thaliana* and pea mutants impaired in this feedback mechanism show an unregulated uptake of TM that leads to an overaccumulation in roots and leaves (19, 20). Moreover, manipulation of TM transporters in a phloem-specific manner leads to delayed transcriptional responses in roots during Fe deficiency experiments, reinforcing the idea that the uptake of TM at the root level is primarily governed by leaves and the levels of TM within the phloem (14). Notably, the xylem vessels typically have a large diameter and a high flow velocity as they are serving the transpiration, while the phloem vessels transport far less volume, thus making phloem sap collection a lot more difficult to obtain and analyze. For this reason, it has not been clearly established whether TM in its ionic form or other more complex molecular interactions with peptides or proteins are required to modulate phloem-driven regulation of TM homeostasis (11).

Due to their reactivity, TM are expected to occur as metal-ligand complexes within cell compartments, including the phloem sap, and not as free ions. In addition, as in any other multi-equilibrium system, the abundance of these metal-ligand complexes depends on several factors, including TM availability, the unique and specific affinity of each ligand for individual TM (*i.e.* dissociation constant), and the pH of each cellular compartment (6, 11). In plants, the nature of TM ligands is diverse, ranging from low molecular weight (LMW) molecules such as organic acids, amino acids, and nicotianamine, to peptides like glutathione (GSH) and phytochelatins, and high molecular weight (HMW) complexes that may include proteins such as metallothioneins (MTs) or LMW complexes organized and assembled as HMW complexes around a stable core (21, 22). In turn, it is expected that cell compartments with different pH and metabolic composition favor specific TM-ligand complexes; however, establishing the chemical speciation of TM in cellular compartments has been a formidable challenge that has been approached almost exclusively by modeling (21, 22, 23). This is due in part to the technical difficulties associated with the extraction and isolation of soluble cell fractions, which often rely on cell disruption techniques leading to a dissociation of native TM-ligand complexes and re-association when compartments with different pH such as the stroma, cytosol, and vacuole are mixed. Here we took advantage of *Brassica napus*, a close relative of Arabidopsis, that has been previously and successfully used to study phloem composition (11, 24). The relatively high volume (hundreds of microliters) of highly pure phloem sap that can be extracted from reproductive tissues, allowed us to conduct a fractionation analysis to determine the HMW/LMW distribution of TM in the phloem sap under native conditions. As expected, none of the TM detected were found as free ions. Glutathione and nicotianamine were also found in LMW and HMW fractions suggesting that in the phloem sap, these LMW complexes may be forming, or stabilizing, HMW complexes. Metallothioneins (MTs) were also found and, unexpectedly, iron (Fe) was found to be associated with MTs. Our results suggest that MTs may play a role in the mobilization of TM other than Cu and Zn and that LMW ligands may help in stabilizing TM-MT complexes in the phloem.

## Results and discussion

**Figure 1:**
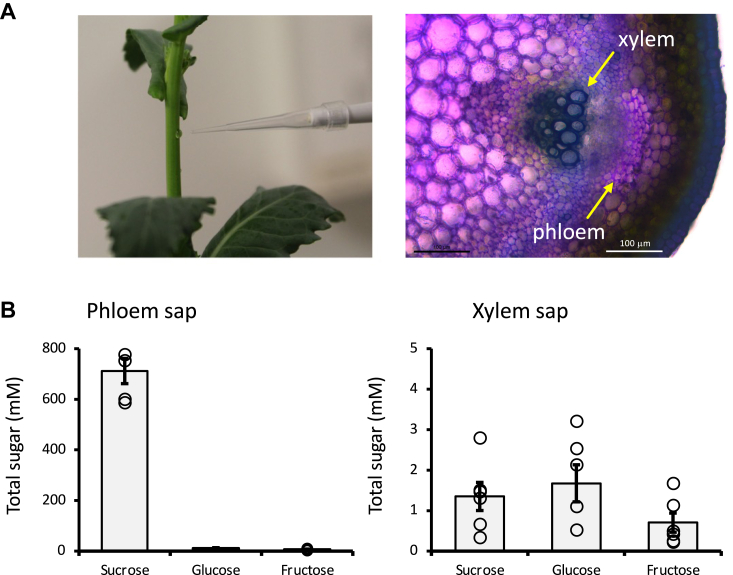
**Phloem sap isolation from *Brassica napus* plants.***A*, sampling near reproductive tissues considering the vascular organization of *B. napus* (*right* panel) allowed us to obtain sufficient quantities of sap for further analyses. *B*, sugar composition of the collected phloem sap (*left* panel), compared to xylem sap sugar content (*right* panel), confirmed the high purity of the phloem sap collection. Data represent mean ± SE of three biological replicates (sap from 3-5 plants were pooled together in each biological replicate).

The phloem sap composition at the proteomic, metabolomic, and ionomic levels has been previously reported for several plant species (11, 24, 25). These studies have advanced our understanding of the basic composition of the phloem sap and contributed to a better understanding of source-to-sink long-distance transport processes. However, due to the difficulty of isolating sufficient quantities of highly pure phloem sap, few attempts have been made to study the phloem sap composition under near-physiological conditions. *Brassica napus* has been previously used to isolate highly pure phloem sap for *-omic* analysis due to its relatively large sieve elements, compared to Arabidopsis, and the known distance between the epidermis and the xylem vessels (Fig. 1*A*). The use of a lancet device allowed us to standardize phloem sap isolation by puncturing the stem at a constant depth. As described before, the first drops were discarded before collecting the sap under a constant stream of nitrogen to minimize oxidation during the sap collection (Fig. 1*B*). To determine the purity of the sap, we quantified the levels of sucrose and the reducing sugars glucose and fructose by High-Performance Anion Exchange chromatography. For comparison, we performed the same measurements on xylem sap samples (*i.e.* apoplastic fluid) obtained from decapitated plants. The high levels of sucrose compared to reducing sugars (>97%) in the phloem sap confirmed its high purity, as this ratio is significantly lower compared to the xylem sap (Fig. 1, *C* and *D*).

### Analytical HPLC-ICP-MS of phloem sap

**Figure 2:**
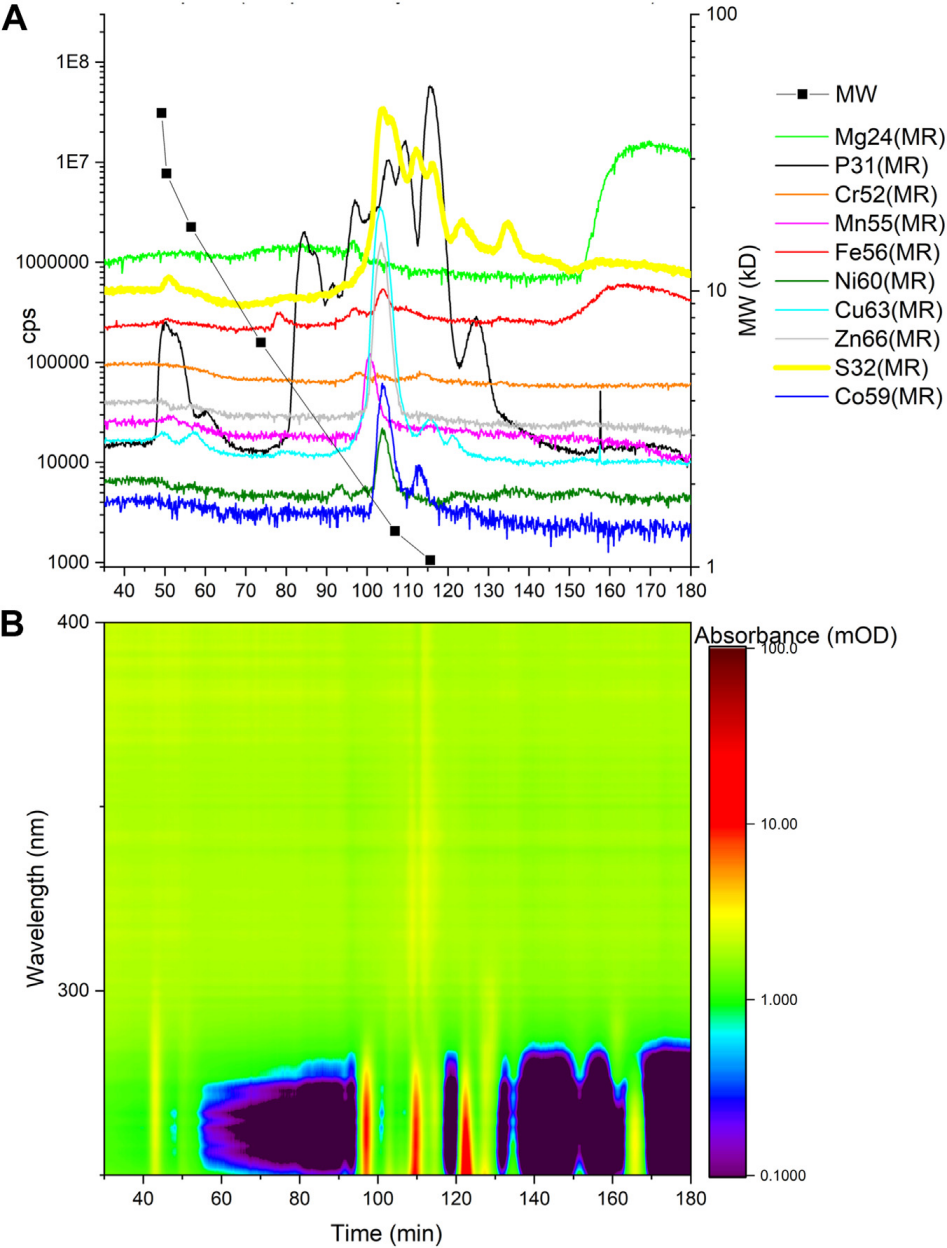
**Phloem sap element speciation under native conditions.***A*, representative chromatogram, selected from three independent replicates, of the native phloem sap *via* size exclusion chromatography (SEC) coupled online to inductively coupled sector field mass spectrometry (ICP-sfMS) and (*B*) the phloem sap diode array spectrometric detection (DAD) spectrum under native conditions. The MW calibration curve shows the following proteins: (1) from Biorad SEC standard: thyroglobulin = 670 kDa, γ-globulin = 158 kDa, ovalbumin = 44 kDa (but all proteins above 40 kDa are not separated any more on these columns optimized for low MW), myoglobin = 17 kDa and vitamin B12 = 1.35 kDa. (2) from the UltraLow gel electrophoresis MW standard from Sigma: Aprotinin from bovine lung (6.5 kDa) and Triosephosphate Isomerase from rabbit muscle (26.6 kD). (3) 4-Aminobenzoic acid 0.137 kDa.

Next, we wanted to fractionate the phloem sap under native anaerobic conditions (*i.e.* pH 7.0) to establish the distribution of TM based on size. Three independent phloem sap samples were analyzed and Figure 2*A* shows a representative HPLC-ICP-MS of the phloem sap and its corresponding multispectral signature (Fig. 2*B*). Most of the metals including Co, Cu, Ni, and Zn were found within one peak at about 1.5 kD, which also contained a high concentration of sulfur (Fig. 2). Mn eluted separately, in one major peak at about 1.8 kD. For iron, the elution pattern had more variation (Fig. 2). For instance, in sample 1, there were 4 Fe-peaks: 5.3kD (medium-sized), 2.2kD (small), 1.5kD (medium-sized), <1kD (the main peak). In sample 2, there were two Fe-peaks, one at about 1.5kD (medium-sized and with some shoulders towards lower MW) and another <1kD (the main peak), and in sample 3, there were 3 Fe-peaks: 3.6kD (very small), 1.5kD (small), <1kD (the main peak). The main very broad “< 1kD” Fe peak, which also contained Mg, eluted at a time beyond the size calibration and is typical for binding interaction with the SEC material as we observed it for trying to run aqueous Fe(II) on the column (not shown). Thus, most of the elements were found in fractions representing molecular weights lower than 10 kDa but none of the TM were found as free ions, which is expected due to their reactivity with proteins and other ligands, or their concentration as free ions was below the limit of detection.

**Figure 3:**
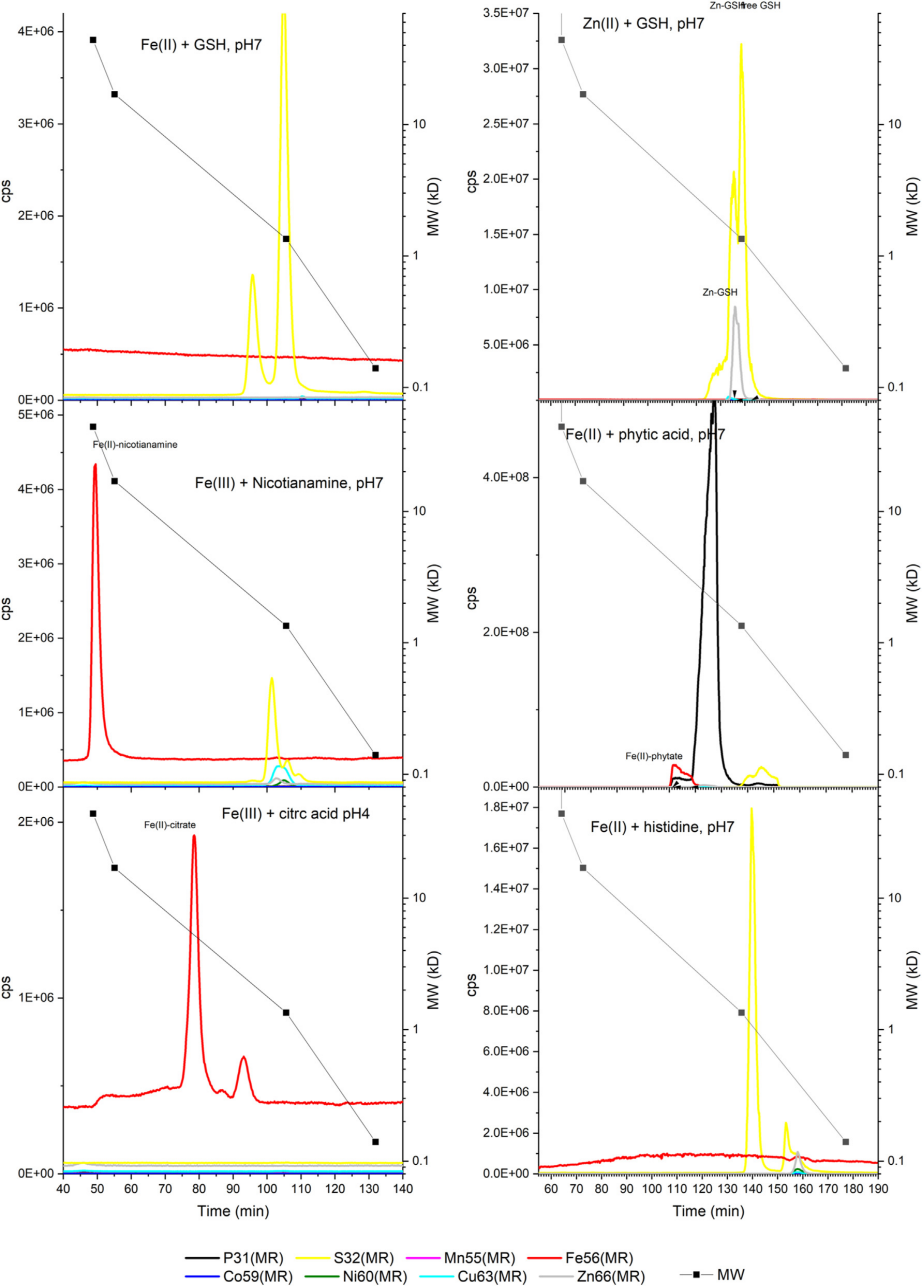
**Selected metal ligand compounds run on the SEC-ICP-sfMS under the same conditions (buffers, pH, flow rate, temperature, column resin) as the phloem sap.** Note that the standards were run on a different set of columns (difference in length, not resin); therefore, the calibrated MW (scale on the right) and not the elution times are to be compared with the chromatograms shown in Figure 2. While this figure is shown in linear scale to clearly show the main contribution, a log scale version is shown as Fig. S1 to show also minor contributions of other elements.

Next, we wanted to understand the behavior of the expected Fe-binding ligands under our chromatographic conditions, either as isolated ligands (*i.e.* GSH) or as the iron(III) complex with either nicotianamine (NA) or citrate (Fig. 3). Surprisingly, NA eluted at a much higher MW than would be expected for a 1:1 complex with iron, eluting at about an apparent MW of 42 kD, while the actual MW of the 1:1 complex should be 360 D where no Fe peak was found. Since no such metal peak was found in phloem sap, it is possible that NA in the phloem sap contributes to maintaining Fe soluble but NA alone may not be the main molecule carrying Fe through the phloem stream. Alternatively, it is possible that at physiological levels, NA complexes in phloem sap aggregate less than the model compound and thus run at lower MW on the SEC. Fe (III)-citrate was found to elute at about 5.1 kD, which corresponds to a minor iron peak in phloem sap sample 1, but was not consistent across samples. Glutathione underwent partial oxidation during the preparation despite all precautions of working in anoxic environments, so both species GSH and GSSG were observed eluting at about 1 kD and 2 kD, respectively. While the Zn-GSH complex could be reconstituted *in vitro*, this did not work for Fe despite starting with Fe(II) and working in a reducing (hydrogen-containing), anoxic atmosphere. As GSH itself has a MW of 0.307 kD, both peaks in the chromatograms resemble tetramers of the respective compounds, and if the main metal peak at about 1.5 kD was indeed GSH complex, suggesting that metals associated with GSH were bound by 4 to 5 GSH ligands. The Fe(II)-phytate complex could be anoxically prepared *in vitro* (Fig. 3), and like sulfur, also phosphorus correlated with metals in phloem sap, but the occurrence of phytate complexes in phloem sap could not be directly confirmed. Notably, Zn(II) and Cu(II) complexes of histidine could be formed *in vitro*, but their apparent molecular weight (0.4 kD) was below the MW of the peaks found in the phloem sap (Fig. 3). Fe-histidine complexes were not found even in *in vitro* preparations. Note that other TM were found in trace amounts while analyzing these standard complexes (Fig. S1), but these were from contaminations of the chemicals from which the standards were prepared, despite the analytical grade used.

**Figure 4:**
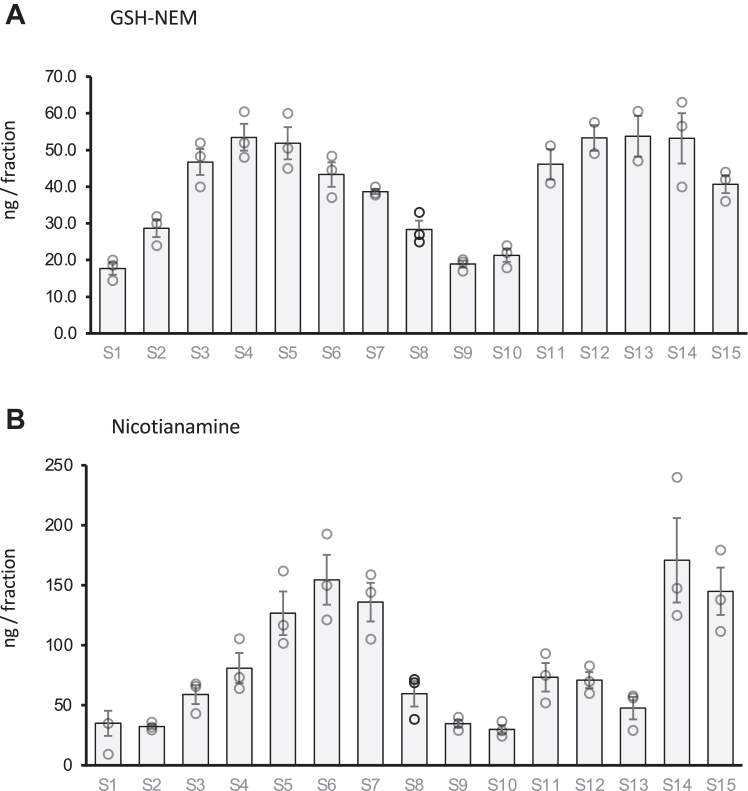
**Glutathione (labeled with NEM) and nicotianamine (NA) measurements in fractions containing the majority of transition elements in the phloem sap.** The main peak around 105 min elution time in Figure 2 was divided into 15 fractions and GSH-NEM (*A*) and NA (*B*) were quantified using LC-MS as described in the methods section. Bars represent mean ± SE of 3 independent phloem sap fractionation.

To begin the identification of TM-binding molecules in the phloem sap, we first focused on the 15 fractions eluting between 95 and 110 min, which contained most of the TM (*i.e.* the main TM peak). We first determined the presence of GSH and NA and found that both molecules were present across these 15 fractions suggesting that these molecules do play a role in the binding and mobilization of TM within the phloem (Fig. 4). The S peak of GSH at about 2 kD agreed well with the main S peak and low molecular weight TM peak, suggesting that GSH plays a role in metal binding and/or stabilization of TM in the phloem sap. In contrast, the results of the NA standard, eluting as an aggregate of about 40 kD, did not match the potential role of NA as the major TM-binding molecule in the phloem sap. Similarly, the small apparent MW of histidine complexes *in vitro* did not match the metal peaks observed in the phloem sap, suggesting that free histidine may play only a minor role in TM phloem transport in non-hyperaccumulator plants like *Brassica napus*.

The occurrence of other metal peaks and high absorbance at 254 nm in the phloem sap chromatograms indicated that further complexes and interactions may be occurring. To gain some insight into TM-metal biding proteins occurring in the sap, we conducted a proteomic analysis of five pooled fractions collected within the main metal peak. Each of these fractions was digested with trypsin and analyzed using a Bruker timsTOF pro mass spectrometer. An initial inspection of the data revealed peptides from 35 different protein groups (Table S1). Some of these peptides, however, belong to proteins with an expected molecular weight higher than the exclusion limit of phloem loading through plasmodesmata (∼35 kDa; (26)). We interpret that these peptides were most likely the result of contamination from neighboring cells when collecting the phloem sap. However, it cannot be entirely excluded that they are also the result of protein degradation and peptide transport through the phloem (27). Within the remaining 19 protein groups, we identified three metallothioneins (MT2, MT3a, and MT3b) present across the five selected fractions. These MTs were consistently identified in three independent phloem sap separations, together with an additional seven proteins whose function has not previously been associated with TM binding capacity (Table 1). The detection of MTs in a low molecular weight peak suggests that these were part of the tail of their main peak or fragments of MTs rather than full-length MTs. However, in all three phloem sap samples, there was a small but significant Cu-Fe-S peak at about 15 kD apparent MW (Fig. 2), suggesting that higher MW complexes may also play a role in the long-distance movement of TM through the phloem.

**Table 1:** The 10 most abundant proteins identified by LC-MS in the main peak containing transition elements in the phloem sap

Accession	Description	Unique peptides (triplicate samples)		
A0A3N6SKZ4|A0A3N6SKZ4_BRACR	Uncharacterized protein OS=*Brassica cretica* OX = 69,181 GN = DY000_00006709 PE = 4 SV = 1	1	2	2
Q852U1|Q852U1_BRAJU	Metallothionein type 3 OS=*Brassica juncea* OX = 3707 GN=BjMT3c PE = 2 SV = 1	1	2	2
A0A3N6TBB2|A0A3N6TBB2_BRACR	Uncharacterized protein OS=*Brassica cretica* OX = 69,181 GN = DY000_00017738 PE = 4 SV = 1	3	5	3
sp|P56170|MT23_BRAJU	Metallothionein-like protein type 2 MT2-22 OS=*Brassica juncea* OX = 3707 PE = 3 SV = 1	7	4	4
sp|P56172|MT25_BRAJU	Metallothionein-like protein type 2 MT2-28 OS=*Brassica juncea* OX = 3707 PE = 3 SV = 1	7	4	4
A0A078K0K2|A0A078K0K2_BRANA	BnaCnng72870D protein OS=*Brassica napus* OX = 3708 GN=BnaCnng72870D PE = 4 SV = 1	3	2	2
A0A078JTM7|A0A078JTM7_BRANA	BnaAnng35580D protein OS=*Brassica napus* OX = 3708 GN=BnaAnng35580D PE = 4 SV = 1	1	2	2
A0A397XTH3|A0A397XTH3_BRACM	Uncharacterized protein OS=*Brassica campestris* OX = 3711 GN=BRARA_I01337 PE = 4 SV = 1	1	1	1
A0A078I8E7|A0A078I8E7_BRANA	BnaA09g19810D protein OS=*Brassica napus* OX = 3708 GN=BnaA09g19810D PE = 4 SV = 1	3	2	2
M4E5L0|M4E5L0_BRARP	Uncharacterized protein OS=*Brassica rapa* subsp. pekinensis OX = 51,351 PE = 4 SV = 1	1	1	1

Fractions containing TE were digested with trypsin and analyzed timsTOF pro mass spectrometer. Only the 10 most abundant proteins present in three independent biological replicates are shown.

### Purification and metal binding properties of native and his-tagged MTs

Next, we investigated the metal-binding properties role of the specific class of MTs found in the phloem sap. Metallothioneins are low molecular weight cysteine-rich proteins that serve various biological functions, including TM homeostasis, detoxification of non-essential elements like cadmium and mercury, and active participation in redox control (28, 29). In plants, MTs have been mostly associated with copper and zinc homeostasis, and global proteomic studies have identified MTs in the phloem sap of different species (29, 30); however, our results suggest that MTs may be binding additional TM in the phloem sap, thus contributing to their long-distance transport.

**Figure 5:**
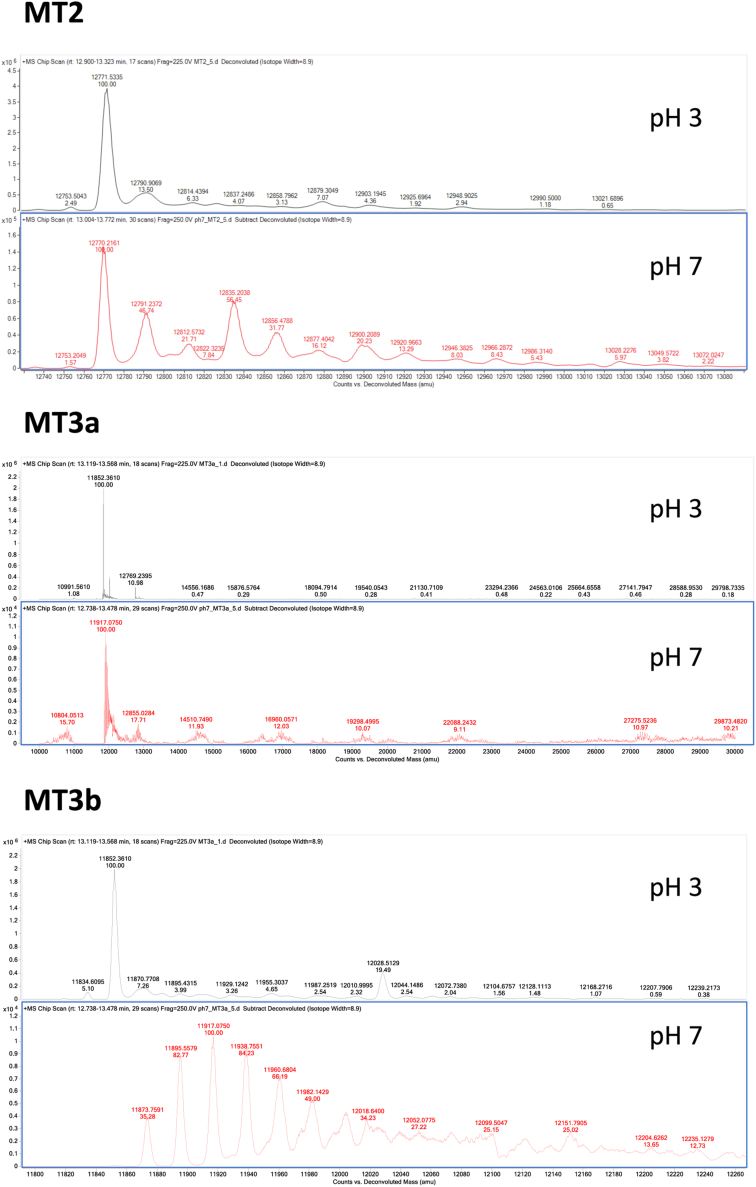
**Intact mass analyses of purified *Brassica napus* metallothioneins at acidic (pH 3, top spectra in each panel) and neutral (pH 7, bottom spectra in each panel) conditions.** Metallothioneins were overexpressed in *E. coli*, purified by affinity-based resins, and analyzed by Quadrupole Time-of-Flight (QToF) mass spectrometry as described in Materials and Methods.

**Figure 6:**
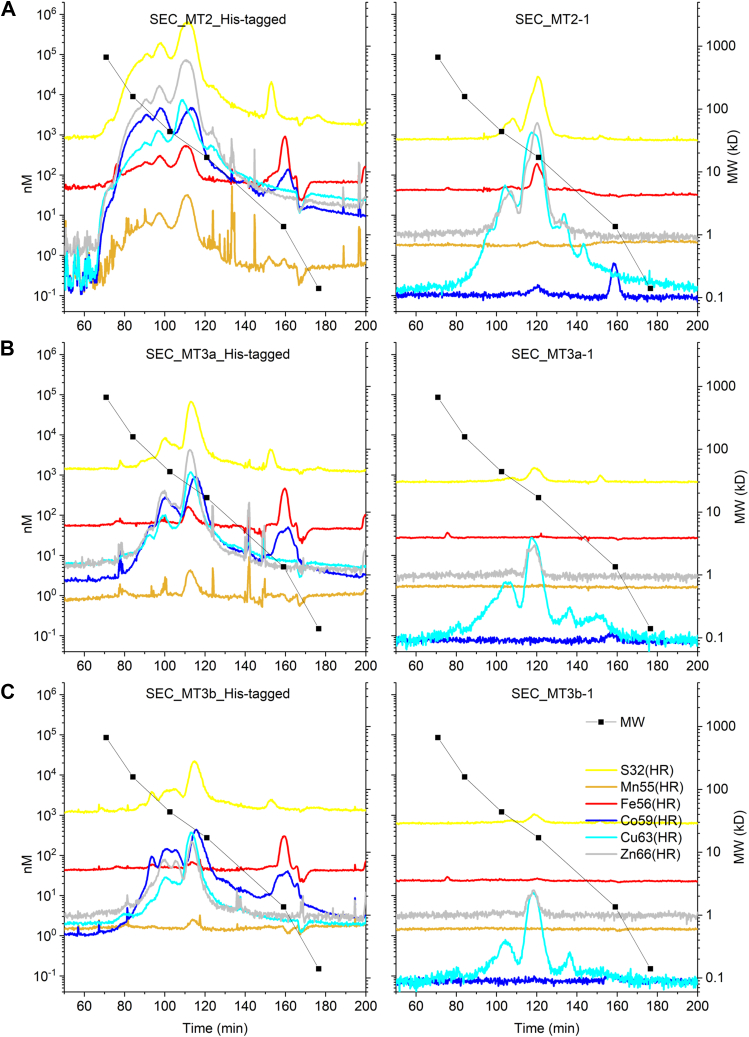
**Metal-binding properties of his-tagged (left panels) and native metallothioneins (right panels) analyzed by size exclusion chromatography (SEC) coupled online to inductively coupled sector field mass spectrometry (ICP-sfMS).** Metallothioneins (A) BnMT2, (B) BnMT3a, and (C) BnMT3b were purified as described in Materials and Methods and were analyzed by SEC-ICP-sfMS. Note that the calibrated MW (scale on the *right*) and not the elution times are to be compared with Figures 2 and 3.

To better understand the metal-binding ability of MTs found in the phloem sap, BnMT2, BnMT3a, and BnMT3b, were cloned, expressed, and purified using *Escherichia coli* as a heterologous expression system. Initially, overexpression was done with an N-terminal 6xHis-tag, as this simplifies protein purification using affinity columns such as a cobalt-based resin. SDS-PAGE and QTOF mass spectrometry analyses confirmed the identity and high purity of the recombinant MTs (Fig. 5). Next, and to gain additional insight on the native structure of these MTs, each intact protein was further analyzed by QTOF at pH 3.0 and pH 7.0 (the physiological pH of the phloem sap. At pH 3.0, the deconvoluted spectra showed the expected mass for each individual protein (Fig. 5). However, species of higher molecular weight (21–300 Da higher) were detected at pH 7.0, consistent with the formation of additional complexes with different elements such as sodium, copper, and likely other elements. Purified MTs were also analyzed by HPLC-ICP-MS (Fig. 6) and several additional features were noticed. For instance, besides the monomeric form of the MTs, various oligomerization forms were found as peaks towards higher MW over a wide range of molecular weights from less than 10 kDa to > 50 kDa. This aggregation was more evident in MT2 than in MT3a/b. In addition, the MT monomers and even more the oligomers were found to have significant amounts of bound cobalt from the metal affinity column, leading to an about equal cobalt:copper ratio in the monomers and more dominant cobalt binding in the oligomers (Fig. 6).

Notably, all 3 MTs were found to bind some iron and manganese, which was at least unusual compared to the literature where MTs are mostly reported to naturally bind Cu and Zn (with individual preferences for one or the other metal). Tagging proteins with a 6xHis tag has the potential to artificially increase the TM binding affinity of proteins, as the purpose of the 6xHis tag is to bind to nickel- or cobalt-containing resins (here we used a Co resin based on the high quality and purity of recombinant proteins obtained compared to nickel-based resins) (31). To systematically address the role of the 6× His tag and the metal binding ability of BnMTs, a second round of overexpression and purification was carried out with non-tagged MTs with subsequent purification *via* anion exchange chromatography, hydrophobic interaction chromatography, and SEC. This native isolation of MTs confirmed that some of the previous observations may be attributed to artifacts caused by the 6× His tag. First, in the native MTs the monomers were more dominant, and while dimers were present, no larger oligomers were found. Second, and perhaps more important, all 3 MTs bound equimolar concentrations of Cu and Zn, but no Co and no Mn. Iron binding, however, was confirmed for MT2, while no Fe was observed for MT3a and MT3b. Iron in the phloem is thought to be bound to low molecular ligands like nicotianamine but previous reports suggested the presence of a “high molecular” protein, but the identity of this protein was not further pursued (32). Notably, MTs in general are considered cytosolic proteins where iron levels are expected to be in the μM range; however, Fe in the phloem can reach sub-millimolar levels (33). Therefore, it is possible that the binding of Fe to MT2 may be the consequence of the unique environment of the phloem and certainly requires more in-depth studies to better understand the TM-binding properties of MTs under different yet physiologically relevant conditions.

**Figure 7:**
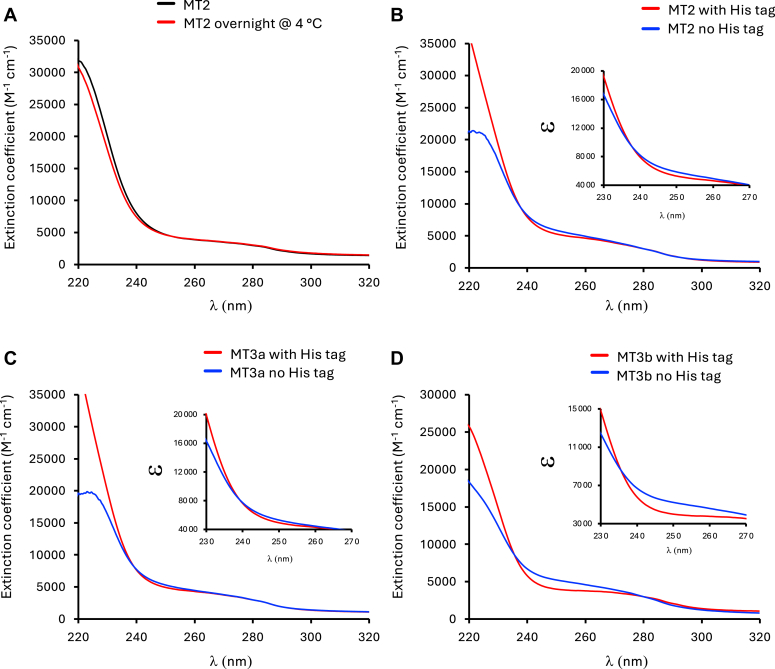
**Absorption spectra of *B. napus* metallothioneins.***A*, overnight incubation at 4 °C has no impact on the spectrophotometric properties of 6xHis-tagged BnMT2. *B-D*, spectrophotometric differences between 6xHis-tagged and tag-cleaved BnMTs. The 6xHis-tag was removed under non-reducing EDTA-free conditions. MT concentration was determined by DTNB titration and plotted using their extinction coefficient (ε). These are representative spectra of at least three independent experiments with similar results.

Studying the metal-binding properties of metalloproteins often requires a significant amount of starting material and the use of 6xHis tags have proven to recover high yields of recombinant proteins. However, our data suggest that the 6xHis tag in MTs interferes with their physicochemical characterization. The removal of 6xHis tags from recombinant proteins can be achieved by including an amino acid sequence that can be recognized by the human rhinovirus (HRV) type 14 3C protease (*e.g.* PreScission protease). This protease typically requires reducing agents such as DTT and metal chelators like EGTA, which may dramatically alter the metal-binding properties of MTs. However, it was recently reported that the HRV-type 14 3C protease can recognize and cleave tags from recombinant proteins under mild conditions; that is, without the need for DTT or EGTA (34). To test whether this approach can be applied to 6xHis-tagged MTs, we conducted some spectroscopy measurements of MTs under different conditions during the purification process. As expected, the high levels of imidazole used to elute recombinant proteins from the Talon affinity have a strong spectral signature that interferes with the characterization of MTs (Fig. S3, *A* and *B*). However, this signature can be efficiently removed by Sephadex G-25 desalting columns. Notably, after the imidazole removal, spectrophotometric scans revealed unique signatures for *B. napus* MT2, MT3a, and MT3b (Fig. S3, *A* and *B*). Next, we tested whether desalting was sufficient to allow for the cleavage of the 6xHis tag using the PreScission protease. We first tested the cleavage of the 6xHis tag using the fluorescent protein mNeonGreen incubated overnight at 4 C in the presence of the protease under non-reducing EGTA-free conditions. The results showed that the 6xHis tag was efficiently removed from mNeonGreen (Fig. S3*C*). We also tested whether an overnight incubation may affect the stability of MTs, and our data show no spectral differences between freshly isolated 6xHis-BnMT2 and the same sample incubated overnight at 4 C (Fig. 7*A*). We then removed the 6x-His tag from all *B. napus* MTs and recorded their corresponding spectrophotometric scans (Figs. 7, *C* and *D* and S3*D*). Interestingly, while each MT retained its own unique spectral signature, there were clear differences between 6x-His tag and non-tagged MTs, particularly at wavelengths < 250 nm. While these results corroborate our previous analyses using non-tagged MTs (Fig. 6), they open the possibility of streamlining the purification of large quantities of tagless MTs for more in-depth metal-binding experiments.

The results of the MT experiments are important in several ways. While the *in vitro* metal binding and size of the native MTs partially resembled their metal binding in the phloem sap, experiments with tag-less MTs were needed to verify this identification. In turn, these data revealed a potential new function of MTs in Cu, Fe, and Zn homeostasis that deserves additional research. In addition, the initial data using His-tag MTs and the discrepancies with tag-less versions of the proteins should serve as a reminder to the scientific community that without proper controls, this seemingly harmless tagging may lead to artifacts. Due to their benefits, His tag proteins can still be used in experiments with the understanding that additional systematic experiments should be conducted to confirm or refine the initial observations. While problems with folding induced by tags (*e.g.* leading to aggregation) are not unknown, the differential metal binding found between tag and non-tagged MTs could have led to wrong conclusions about these metalloproteins.

## Conclusion

Here we report the fractionation and characterization of the phloem sap of *Brassica napus* grown under standard soil conditions (*i.e.* replete nutrient availability) and present two important conclusions related to metal speciation in the phloem sap and the effects of his-tagging on metalloproteins. For the phloem sap, our data recapitulated previous reports in terms of the proteomic and metabolomic composition of phloem sap but we also discovered an unexpected behavior of well-known low and high molecular weight TM ligands. That is, MTs were found in the phloem as different molecular species, and low molecular ligands such as GSH co-purify with MTs. Our data suggest that the unique environment of the phloem sap and its composition may be conducive to the formation of previously unknown complexes containing MTs, TM, and GSH. The phloem plays critical roles in nutrient allocation and nutrient signaling, yet phloem sap biochemistry remains largely unexplored and clearly requires additional investigation. As for protein tagging, the main conclusion is that the native untagged expression should be the “gold standard” for overexpressing metalloproteins; and if tags are used, they should be carefully chosen to prevent artifacts related to the metal binding properties of metalloproteins.

## Experimental procedures

*Brassica napus* L. cv Westar was grown in a growth chamber 16/8 h light/dark conditions at 23 °C day/21 °C night, and the humidity was maintained at 60%. Flowering plants were used for phloem collection as described before (11) under a gentle nitrogen stream to reduce the levels of oxygen around the collection point. The purity of phloem sap was determined by quantifying sucrose, fructose, and glucose ratios using High-Performance Anion Exchange (HPAE) chromatography analysis (35).

### Analytical and semi-preparative HPLC-ICP-MS

#### Buffer preparation

Water used for buffers and preparation of standards was first purified by reverse osmosis and filtering (Demiwa 20 rosa, Watek, Ledeč N.S), afterward double distilled (Destamat bi18e, QCS), and subsequently referred to as roddH_2_O. This provided the lowest background level of metals that could be achieved with current technology. All the labware was acid-washed in 5% HNO_3_ and afterward with roddH_2_O before use. Wherever possible, PFA labware was used. Chemicals used in the preparation of buffers were of ultra- or supra-pure grade whenever available: Ammonium bicarbonate and Ammonium acetate were “BioUltra” grade (Merck KGaA). Nevertheless, buffers still had a too high background of iron, therefore it were additionally batch-treated with Chelex-100 (Biorad) that was regenerated with roddH_2_O and “Suprapur” grade KOH (Merck KGaA).

#### Configuration of the HPLC-ICP-MS system

Both, the analytical separation of metal complexes in phloem sap and protein extracts from MT overexpression in *E. coli*, and the semi-preparative purification of MTs from their non-tagged overexpression in bacteria were performed with a customized metal-free HPLC system with desolvating injection to sector-field ICP-MS as described by (36). In brief, a customized version of the Azura 6.1 L system (Knauer GmbH) was used, in which the complete flow path was free of metallic surfaces in order to minimize metal contamination of the mobile phase. This was achieved by selecting ceramic pump heads, valves, and tubes made of ceramics and/or PEEK, and a flow cell in the diode array detector made of PEEK + quartz. A customized version of the Element XR (Thermo Fisher Scientific) inductively coupled plasma sector-field mass spectrometer (ICP-sfMS), in which a “jet interface” provided increased sensitivity was used, for aqueous samples together with a desolvating nebulizer (Apex Q). The instrument was optimally tuned to reduce the potential interferences by choosing medium resolution (4000) or high resolution (10,000) with an acceptably low oxide formation rate as monitored by CeO^+^/Ce^+^. The typical operating conditions of the ICP-sfMS Element XR-2 were as follows: RF power, 1250 W; oxide ratio CeO^+^/Ce^+^, 1.0 − 1.2%; doubly charged Ce^2+^/Ce^+^, 1.0 − 1.2%; auxiliary gas, 80 L/min; sample gas flow, 1.20 L/min (variable); cool gas, 16 L/min; extraction lenses, −2000 V; medium resolution, 4000, high resolution 10,000. The details of the method optimization of HPLC-ICP-MS are described in (36).

#### HPLC-ICP MS conditions for phloem sap analysis

For these analytical separations, high-resolution size exclusion chromatography with optimization for small peptides was applied. To optimize resolution, three columns were run in series: a self-packed 10 × 600 mm and two pre-packed Superdex30 Increase columns (GE Healthcare). A gel filtration calibration standard (Bio-Rad, containing thyroglobulin = 670 kDa, γ-globulin = 158 kDa, ovalbumin = 44 kDa, myoglobin = 17 kDa, and vitamin B12 = 1.35 kDa) was used to determine the size and molecular weight of our proteins of interest, 4-Aminobenzoic acid (=para-aminobenzoic acid, PABA) was added to it to extend the calibration to 0.137 kDa. In addition, and different from later work, MW standard curve of these analyses contains the following proteins. (1) from the UltraLow MW standard from Sigma: Aprotinin from bovine lung (6.5 kDa) and Triosephosphate Isomerase from rabbit muscle (26.6 kD). We did not use it later because the other proteins it contains do not produce a useful UV/VIS absorption signal in the DAD, and in later work, we only used the BioRad SEC standard with added PABA. For the analysis, frozen phloem sap was thawed in an anoxic glovebox (atmosphere of 98% nitrogen with approx. 2% hydrogen) and ultracentrifuged in this anoxic state at 100,000*g* for 10 min to remove any insoluble/precipitated particles (none were observed). Afterward, 50 μl of sap was injected into the HPLC, where the buffer was kept anoxic by bubbling with N_2_. All further running conditions were as described by (36).

#### HPLC-ICPMS conditions for semi-preparative purification of MTs

For this purpose, the SEC separation was preceded by anion exchange chromatography (AEC) and hydrophobic interaction chromatography (HIC), both with a buffer system and column combination optimized for their coupling to the ICP-MS, as described in detail in a separate methods publication (Küpper *et al.*, 2024, submitted). In brief, due to their requirement for high-salt buffers, these methods are classically incompatible with ICP-MS, which we eliminated by using completely volatile ammonia-based compounds as salts and which furthermore minimized the flow to the ICP-MS. Columns and gradients were optimized for maximal resolution. Otherwise, the running conditions were as described by (36). The purification was started with anoxic isolation and handling (using otherwise the same isolation procedure as described by (4)) followed by anoxic injection to either the SEC or the AEC that were running with anoxic buffer. In both cases, the fractions of Fe, Cu, and/or Zn-containing peaks were collected and re-injected to HIC. The HIC fractions of Fe, Cu, and/or Zn-containing peaks were collected again, and re-injected to SEC for the purification started with AEC, or to AEC for the purification started with SEC. In this way, we could verify that the later, oxic preparation steps led to the same metal complement and aggregation state of the MT’s as detected in samples that were directly, anoxically injected into SEC.

#### Synthesis and HPLC-ICPMS conditions for model complexes

The model complexes were prepared by adding an excess of metal to the free ligand under conditions that are known to favor complex formation. This means a 10-fold excess of the ligand was used, except for NA where only a 1.2-fold excess of the ligand was used. Generally, the complexes were prepared in the buffer used for SEC. For glutathione and generally, for Fe(II) containing complexes, all preparation work was done with buffers made anoxic by bubbling with pure nitrogen gas and handling in a glovebox with N_2_/H_2_ (forming gas) atmosphere. Furthermore, sodium dithionite was added as a reductant to keep the Fe(II) redox state during complex formation. For the SEC, like for the phloem sap Superdex30 columns were used. While originally one column of Superdex30 prep (10 × 600 mm, see above) coupled to one Superdex30Increase (10 × 300 mm) was used, later a second Superdex30Increase (10 × 300 mm) was added to further improve resolution.

### Proteomics

Phloem samples were dried and re-suspended in urea buffer and digested with trypsin. Digested peptides were purified using C18 tips and lyophilized. The samples were then resuspended in 5% Acetonitrile and 0.1% Formic acid. 2ul suspended peptide was separated on the C18 column (20 cm × 75 μm 1.7 μm) with a gradient of acetonitrile at 400 nl/min. The Bruker nanoElute system is connected to a timsTOF pro mass spectrometer. LC gradient conditions: Initial conditions were 3%B (A: 0.1% formic acid in water, B: 99.9% acetonitrile, 0.1% formic acid), followed by a 14.5 min ramp to 30%B. 30 to 50%B over 2 min, gradient of 50%B to 80%B over 1.5 min, hold at 80%B for 7 min. Total run time was 25 min. MS data were collected over a m/z range of 100 to 1700. During MS/MS data collection, each TIMS cycle included 1 MS + an average of 10 PASEF MS/MS scans. The acquired data were submitted to the PEAKs (version x) search engine for protein identification. Data were searched with trypsin as enzyme (unspecific), two missed cleavages allowed; carbamidomethyl cysteine as a fixed modification; oxidized methionine and acetylation on protein N terminus as variable mod; 50 ppm mass tolerance on precursor ions, 0.1 Da on fragment ions. Uniprot brassica (Swiss-Prot and TrEMBL) protein database was used. For intact mass measurements, samples were analyzed at pH 3.0 and pH 7.0 using an Agilent Technologies 6520A QTOF. Samples (2uL) were loaded in sequence as follows: blank (5% acetonitrile 0.1% formic acid), sample, and then an additional blank. Data were then examined using the qualitative analysis software provided with the instrument. The Maximum entropy algorithm was used to deconvolute multi-charge-state peaks to intact protein mass using the following parameters: mass: 10,000 to 20,000 Da; mass step: 1 Da; m/z range restricted: 700 to 3200; baseline subtraction factor: seven; peak s:n of 30; 25% peak height for mass averaging; minimum charge state required for averaging: five; iterations: 40.

### Metabolomics

GSH, GSSG, and nicotianamine (NA) were determined by multiple reaction monitoring (MRM) using a UPLC tandem quadrupole mass spectrometer (Xevo TQS, Waters) operated in positive mode (Fig. S2). Each method was established using analytical standards and direct infusion of GSH, GSSG, and NA standards (5ug/ml, in 50% CAN, 0.1% formic acid) into the mass spectrometer at a flow rate of 20 μl/min. Samples (10 μl injection) were separated using an HSS T3 (Waters) C18 column (1.7um, 10 cm × 2.1 mm i.d.) by gradient delivery (0.4 ml/min) of solvent. Solvent A: 0.1% formic acid in water. Solvent B: 0.01% formic acid in acetonitrile. Initial conditions were 0.1%B followed by a 2 min ramp to 2%B, 1.5 min gradient to 30%B, 0.1 min ramp to 95%B, hold at 95%B for 1 min, 0.1 min ramp to 0.1%B, and hold at 0.1%B for 0.8 min. The total run time was 5.5 min. The column was heated to 45 °C and the samples were cooled to 10 °C in the autosampler. The MRM transitions were as follows: GSH (308.1 > 162.07; 308.1 > 179.11), GSSG (613.42 > 355.19; 613.42 > 231.09) and NA (304.26 > 185.22; 304.26 > 114.04).

### Heterologous expression, purification of 6xHis tagged proteins, and spectrophotometric characterization

*Brassica napus* metallothioneins MT2, MT3a, and MT3b carrying a PreScission Protease before the start codon was synthesized by Genscript (Genscript Biotech Corp) flanked by Gateway attachment site to facilitate subcloning by LR Clonase II recombination. Each MT was then recombined into pET-DEST42 destination vector following the manufacturer's recommendations (Invitrogen, ThermoFisher). Sequences were confirmed by Sanger sequencing before being transformed into *E. coli* Rosetta 2 (pLysS) cells for overexpression and purification as previously described (31). To generate MTs without 6xHis tags, the coding sequence of MTs was amplified using primers listed in Table S2 and ligated back to pET-DEST42, previously digested with SacI and SalI, using the In-Fusion cloning system (Takara Bio). To remove the 6xHis tag under non-reducing and EDTA-free conditions, purified 6xHis-tagged MTs were first desalted using Sephadex G-25 equilibrated with 120 mM KCl, 50 mM Tris (pH 8.0), and the volume was adjustewd to 2.0 ml. Then, 10 μl of PreScission Protease (Genscript Biotech Corp) was added to each 6xHis-tagged MTs and the mixture was incubated overnight at 4 C. The following day, the solution was passed through 1 ml of Talon affinity resin (Takara Bio) equilibrated with 120 mM KCl, 50 mM Tris (pH 8.0) and the flowthrough was collected. The concentration of each MTs was determined by titrating each MT with DTNB as described in (37) and plotted using a theworetical ε 2980 M^−1^ cm^−1^. MT spectra were recorded using a Jasco V-750 spectrophotometer (Jasco, Inc) from 220 to 420 nm at a rate of 100 nm/min.

## Data availability

The list of all proteins identified by mass spectrometry is included as Supporting information. Raw chromatograms from the inductively coupled plasma sector-field mass spectrometer (ICP-sfMS) as well as bacterial strains and plasmids are available upon request. Please contact the corresponding authors if needed.

## Supporting Information

Additional tables, figures, and files accompanying this manuscript are available as Supporting Information.

## Conflict of interest

The authors declare that they have no conflicts of interest with the contents of this article.
