# Supplementary material for: Identification and characterization of transition metal-binding proteins and metabolites in the phloem sap of Brassica napus
Source: J Biol Chem. 2024 Aug 31;300(10):107741. doi: 10.1016/j.jbc.2024.107741 (PMC11497405; doi:10.1016/j.jbc.2024.107741)
Supplement: Supporting Figures [file mmc3.pdf]

Supporting Information

This manuscript contains two supporting tables, three supporting figures, and 3 gene sequences, one for each metallothionein.

Supporting Table 1. All proteins detected by LC-MS in the phloem sap of Brassica napus. Protein groups refer to proteins that cannot be unambiguously identified by unique peptides but by shared peptides, thus these proteins are grouped into one protein group and quantified together. This is a standard procedure in proteomic analyses.

Supporting Table 2. Primers used to re-clone *B. napus* metallothioneins without a 6xHis tag.

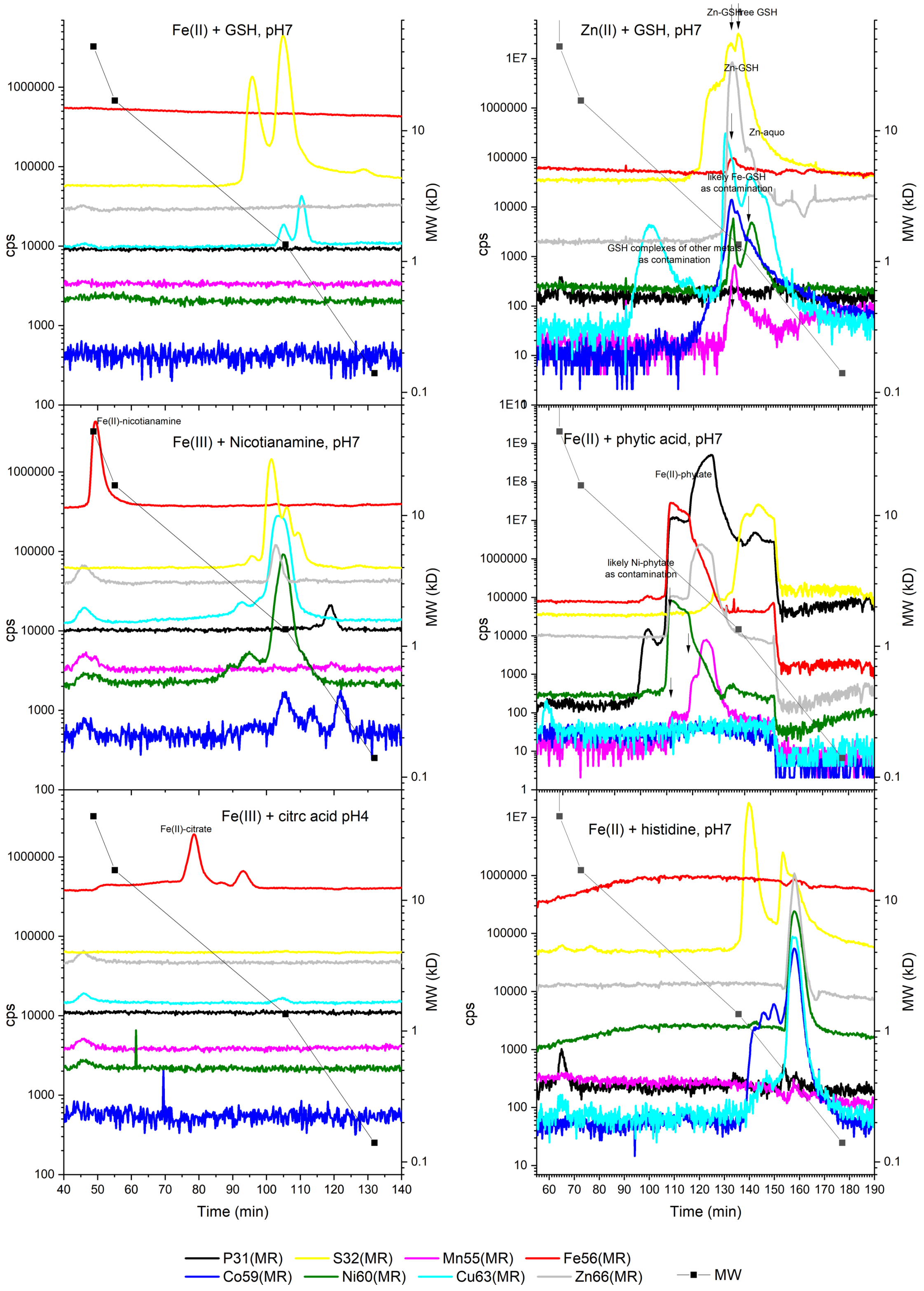

Supporting Figure 1 Log scale version of Figure 3, showing also minor contributions of TM. In this way, multiple peaks with multiple TM could be detected, which are very small contributions, as seen when showing the main metal in linear scale (Figure 3 main manuscript). Thus, the additional peaks/metals are considered background, and this level of *contamination* is inevitable with all commercially available chemicals, even when using the highest purity available in the market as used in this study.

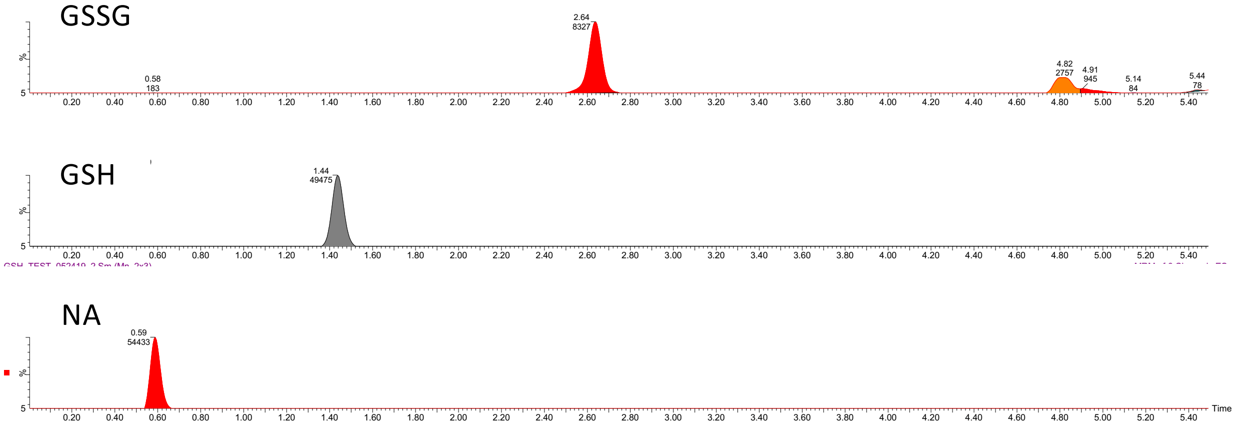

Supporting Figure 2 GSH, GSSG and nicotianamine (NA) were determined by multiple reaction monitoring (MRM) using a UPLC tandem quadrupole mass spectrometer as described in Experimental Procedures.

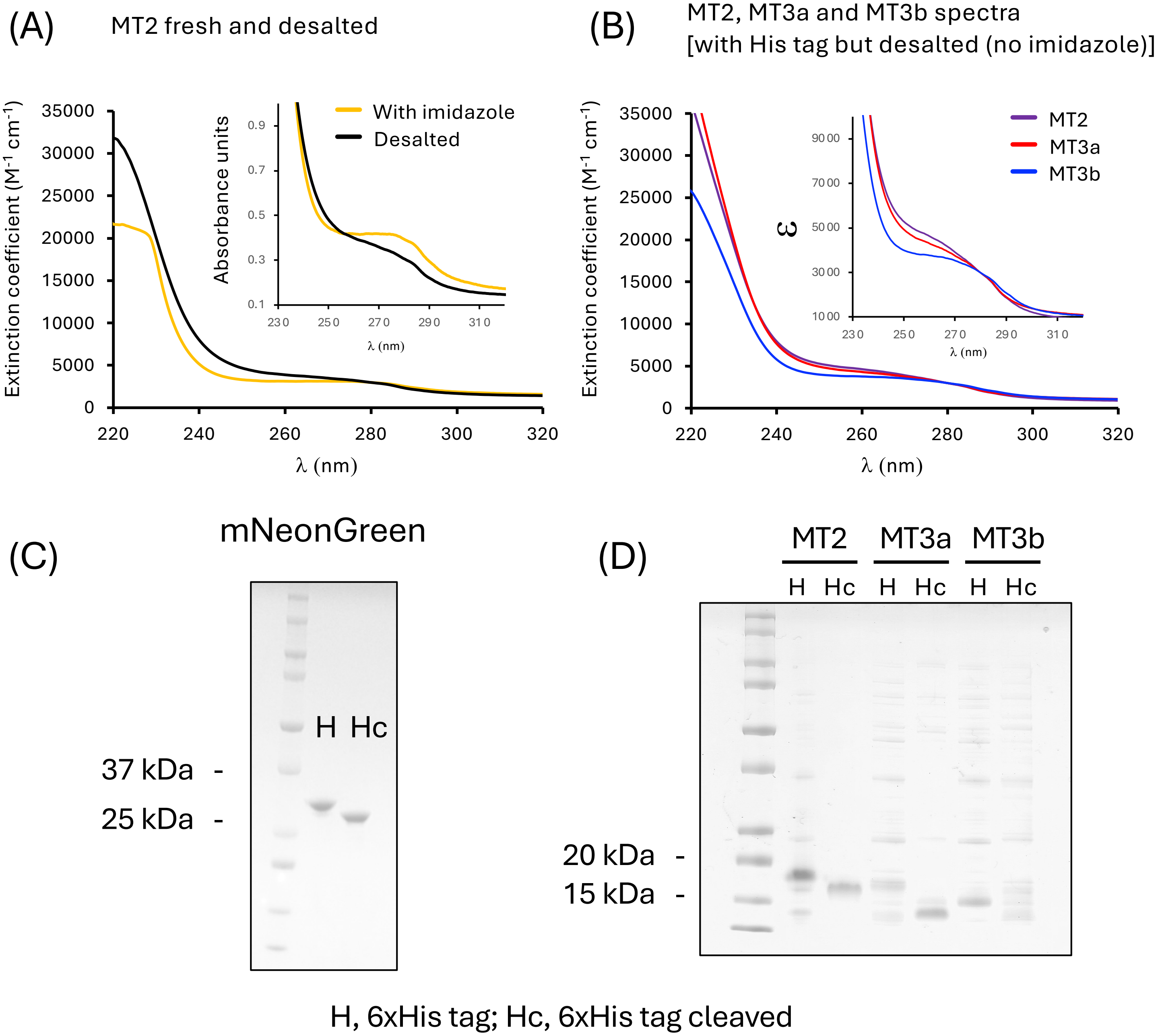

Supporting Figure 3. Removal of the 6xHis tag under non-reducing and EDTA-free conditions. (A) His-tagged proteins eluted with 200 mM of imidazole from the Talon resin show abnormal spectroscopy properties that may interfere with further experiments including 6xHis tag removal. (B) 6xHis-tagged metallothioneins show unique spectral properties after removing the imidazole using a Sephadex-G25 resin (C) Desalting 6xHis tagged proteins is sufficient to allow an efficient cleavage of the 6xHis tag using a PreScission Protease without the need to add reducing agents or EDTA. This procedure was first established using a 6xHis tagged mNeonGreen for easy tracking of tagged/non-tagged proteins (D) The same procedure allowed the removal of the 6x-His tag from recombinant *Brassica napus* metallothioneins.
