# Supplementary material for: Identification and characterization of transition metal-binding proteins and metabolites in the phloem sap of Brassica napus
Source: J Biol Chem. 2024 Aug 31;300(10):107741. doi: 10.1016/j.jbc.2024.107741 (PMC11497405; doi:10.1016/j.jbc.2024.107741)
Supplement: Supporting Information [file mmc4.docx]

**Supporting information**

Sequence used for gene synthesis from the start codon to the stop codon. The 6xHis tag is highlighted, that PreScission Protease site is underlined and the native ATG of each metallothionein is shown in bold.

> Presc_BnMT2

ATGGCACATCACCACCACCATCACGTGGGTACCGGTTCGAATGATGACGACGACAAGAGTCCGGATCCAATGCTGGAAGTTCTGTTCCAGGGGCCC**ATG**TCTTGCTGTGGAGGAAACTGTGGTTGCGGAGCTGGCTGCAAGTGCGTTGGATGCGGAGGTTGCAAAATGTACCCAGACTTGAGCTTCTCCGGCGAGACCACCACCACCGAGACTCTTGTCCTCGGCGTTGCTCCGTCGATGAACTCTCAGTACGAGGCTTCCGGCGAGACTTTCGTCGCCGAGAATGATGCCTGCAAATGCGGATCTGACTGCAAGTGCAACCCTTGCACCTGCAAATAG

> Presc_BnMT3a

ATGGCACATCACCACCACCATCACGTGGGTACCGGTTCGAATGATGACGACGACAAGAGTCCGGATCCAATGCTGGAAGTTCTGTTCCAGGGGCCC**ATG**TCTTCGTGCGGAAACTGCGACTGTGCTGACAAGACCCAGTGCGTGAAGAAGGGAACCAGCTACACCTTCGACATCGTCGAGACTCAGGAGAGCTACAAGGAAGCCATGATCATGGACGTTAATGGTGCAGAAGAGAACGGGTGCCAATGCAAGTGTGGCTCTAGCTGCAGCTGCGTCAACTGCACTTGCTGCCCCAATTAA

> Presc_BnMT3b

ATGGCACATCACCACCACCATCACGTGGGTACCGGTTCGAATGATGACGACGACAAGAGTCCGGATCCAATGCTGGAAGTTCTGTTCCAGGGGCCC**ATG**TCGGACAAGTGCGGAAGCTGCGACTGTGCTGACAAGACCCAGTGCGTGAAGAAGGGAACCAGCTACATCTTGGACATCATCGAGACTCAGGAGAGCTACAAGGAAGCCATGTTCATGGACGTTGGTGCAGAAGAGAACGGGTGCCAATGCAAGTGTGGATCTACCTGCAGTTGCGTCAACTGCACTTGCTGTTAA
